# Defensive Symbioses in Social Insects Can Inform Human Health and Agriculture

**DOI:** 10.3389/fmicb.2020.00076

**Published:** 2020-02-07

**Authors:** Jennifer R. Bratburd, Rachel A. Arango, Heidi A. Horn

**Affiliations:** ^1^Department of Bacteriology, University of Wisconsin-Madison, Madison, WI, United States; ^2^Forest Products Laboratory, United States Forest Service, United States Department of Agriculture, Madison, WI, United States

**Keywords:** defensive symbiosis, social insects and humans, gut microbiome, colonization resistance, model systems, social immunity, insect agriculture

## Abstract

Social animals are among the most successful organisms on the planet and derive many benefits from living in groups, including facilitating the evolution of agriculture. However, living in groups increases the risk of disease transmission in social animals themselves and the cultivated crops upon which they obligately depend. Social insects offer an interesting model to compare to human societies, in terms of how insects manage disease within their societies and with their agricultural symbionts. As living in large groups can help the spread of beneficial microbes as well as pathogens, we examine the role of defensive microbial symbionts in protecting the host from pathogens. We further explore how beneficial microbes may influence other pathogen defenses including behavioral and immune responses, and how we can use insect systems as models to inform on issues relating to human health and agriculture.

## Introduction

**FIGURE 1:**
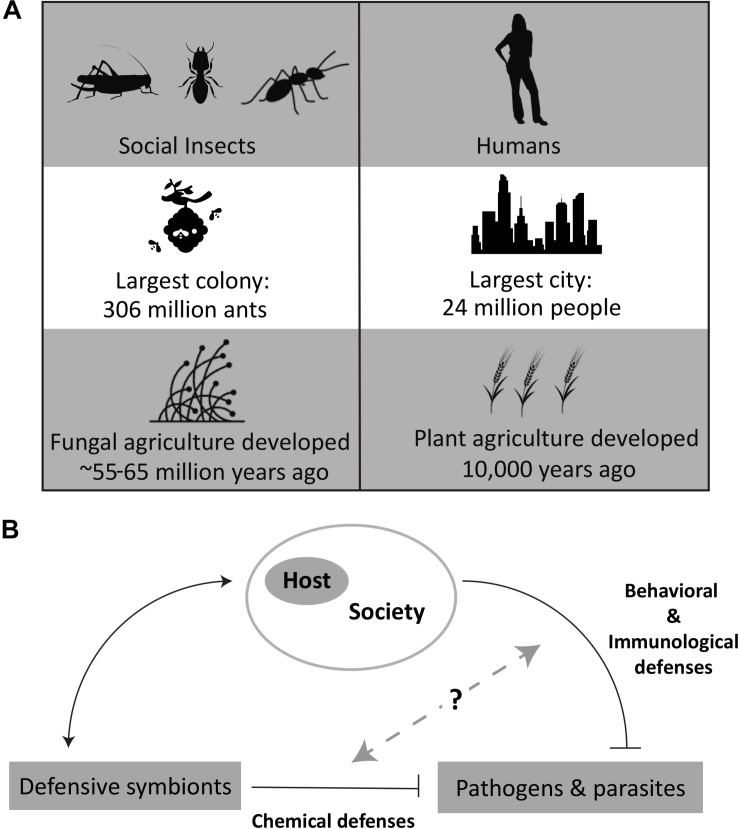
**(A)** Comparisons of human and insect societies, based on social grouping sizes (Burchill and Moreau, 2016; Sawe, 2018) and history with agriculture (Pringle, 1998; Schultz and Brady, 2008). **(B)** Overview of the relationship of defensive symbionts with host and pathogens. Specific image credit from the Noun Project (https://thenounproject.com/): Woman by Lluisa Iborra, Locust by OCHA Visual, Termite by Heberti Almeida, Ant by Jacob Eckert, City by sumhi_icon, Beehive by Juraj Sedlák, Barley by Nathan Stang, and Fungi by CombineDesign. All images used and modified under the Creative Commons License, Attribution 3.0.

Some of the most successful species on the planet in terms of number of species generated over time, ability to inhabit diverse ecosystems, and maintenance of high population densities are social animals (Wilson, 1987). Social lifestyles, however, come at the cost of increased exposure to pathogens. Both modeling and experimental results indicate that population size and density correlate with pathogen prevalence and diversity (Anderson and May, 1979, 1982; Altizer et al., 2003; Schmid-Hempel, 2017). The 10-fold expansion of the human population in the last 200 years with similar population density increases has caused concerns around the risk of spreading infectious diseases (Cohen, 2003). Social insects have faced the same challenges successfully, maintaining high population densities over millions of years and are simple models to gain a better understanding of how to mitigate pathogen burden and spread (Figure 1).

While social living may enhance pathogen spread, social living also enables the spread of beneficial microbes (Biedermann and Rohlfs, 2017). For instance, after termites molt, they must replace their gut symbionts from other nest mates through trophallaxis and coprophagy. This “social gut” is suggested to contribute to nestmate recognition as well as development, nutrition, and defense (Breznak and Brune, 1994; Matsuura, 2001; Nakashima et al., 2002; Adams and Boopathy, 2005). Many microbes benefit the host by providing protection against predators, parasites, pathogens, or environmental stresses, also known as defensive symbiosis (White and Torres, 2009). In a mutualistic relationship, the host provides shelter and/or nutrients in exchange for defense. Understanding interactions between hosts, pathogens, and beneficial microbes can inform on the potential use of beneficial symbionts in systematically targeting certain pathogens.

In interactions between social animals, their microbial defensive symbionts and pathogens, many different selective pressures may be operating simultaneously. Pathogen pressures can impact host and symbiont (King and Bonsall, 2017; Engl et al., 2018). Beneficial symbionts may influence social behavior to facilitate their horizontal transmission, but core microbiota may be influenced by diet or other factors (Sherwin et al., 2019). The evolutionary and ecological dynamics of microbial symbiont relationships with social animals are not well understood. To deconvolute these interactions, social insects are interesting models to compare social and solitary relatives (e.g., bees, discussed below) or comparing changes in microbiota of species that alternate between gregarious and solitary lifestages may also be useful (Lavy et al., 2018).

In this review, we discuss the role of microbial defensive symbionts in pathogen mitigation within social communities and their associated agricultural systems. We also consider how defensive symbiosis intersects with immunological and behavioral defenses. We compare examples from insects with defensive symbionts in humans and highlight how insect models can advance understanding the social impacts of defensive symbionts.

## Insect Defenses Against Pathogens

While defensive symbionts can benefit both social and solitary animals, social living may better enable sharing defensive symbionts than solitary lifestyles. For example, eusocial bees (e.g., *Apis mellifera* and *Bombus* spp.), have a consistent core microbiota that defends against the trypanosome gut parasite *Crithidia bombi*, whereas solitary bees do not have a consistent core community (Koch and Schmid-Hempel, 2011). Several core microbiome members, including *Gilliamella apicola* and *Lactobacillus* spp., correlate with decreased susceptibility to *C. bombi* (Cariveau et al., 2014; Mockler et al., 2018; Näpflin and Schmid-Hempel, 2018). Additionally, experiments disrupting the core bee microbiota support the hypothesis that the gut microbiota plays a role in protecting against opportunistic pathogens (Raymann et al., 2017) and another common parasite, *Lotmaria passim* (Schwarz et al., 2016). Biofilm formation by the core strains is the suggested protective mechanism against this pathogen, as indicated by fluorescent *in situ* hybridization (FISH) imaging (Martinson et al., 2012) and the enrichment of secretion systems and surface proteins in bee gut metagenomes (Engel et al., 2012). As biofilm formation and colonization resistance are broad defensive mechanisms, it is unclear whether solitary bees have microbes with similar functionality. Likewise, social bee gut microbes may confer other functions affecting fitness.

Social animals need to not only protect themselves from disease, but also their shared food sources. Three lineages of eusocial or subsocial insects demonstrate agricultural behavior: ants (Myrmicinae: Attini), termites (Macrotermitinae), and ambrosia beetles (Xyleborinae and others). All of these insects live in gregarious communities supporting the hypothesis that sociality allowed for evolution of insect agriculture (Mueller et al., 2005). Fungus farming termites cultivate basidiomycete fungi, *Termitomyces* spp. as a food source that are either vertically or horizontally acquired depending on termite species (Johnson and Hagen, 1981; Korb and Aanen, 2003). Some termites (*Macrotermes natalensis*) harbor *Bacillus* sp. that produce bacillaene which has antifungal activity and helps protect the fungal cultivar (Um et al., 2013). Xyleborine ambrosia beetles cultivate an assemblage of fungi, rather than a single fungal cultivar, which comprises mycelial fungi, yeasts, and bacteria (Norris, 1965; Hulcr and Stelinski, 2017). A cycloheximide-producing *Streptomyces* phylotype has been isolated from two species of ambrosia beetles as a possible defensive symbiont (Grubbs et al., 2019).

In the fungus-growing ants, microbial associations range from mutualistic to parasitic and are well-described. The ants grow a fungal cultivar as their primary food source in a monoculture, which makes it highly susceptible to the specialized fungal pathogen *Escovopsis* (Ascomycete; Hypocreales). To protect their food source, the ants evolved several defense mechanisms, including a mutualism with *Pseudonocardia* spp. (Currie et al., 1999b, 2003). *Pseudonocardia* produces antimicrobial molecules that are active against *Escovopsis* (Currie et al., 1999b, 2003; Poulsen et al., 2010). Growing *Pseudonocardia* and *Escovopsis* together reveals patterns of inhibition and resistance between the two organisms suggesting population and interaction dynamics at fine phylogenetic scales (Poulsen et al., 2010; Cafaro et al., 2011). Several of the antibiotics produced by *Pseudonocardia* have been characterized (Oh et al., 2009; Carr et al., 2012; Van Arnam et al., 2016) although the full diversity of antibiotics used is unknown.

## Interactions of Defensive Symbionts With Host Defenses in Insects

Other methods of pathogen resistance, such as behavior and immunity, aid in disease resistance and can be influenced by microbes (Nyholm and Graf, 2012; Lizé et al., 2014; Flórez et al., 2015). Host and symbionts may adapt to each other in different ways: symbionts may avoid triggering immune function (Trappeniers et al., 2019); hosts may diversify immune pathways (Maire et al., 2019) or hosts may potentially reduce immune function (International Aphid Genomics Consortium, 2010; Douglas et al., 2011). Further examples of innate immunity in social insects can be found in the following review (Otani et al., 2016).

Social insects can coordinate defensive behaviors, some of which may be triggered or helped by beneficial microbes. Many of the defensive behaviors in social insects are aimed at maintaining sanitation of the nest as well as the individuals within the nest. This phenomenon of collective actions to mitigate pathogen spread/exposure is known as social immunity, which is defined as the control or elimination of potential pathogens by cooperation of individuals through behavioral, physiological, and/or organizational means (Cremer et al., 2007; Meunier, 2015). For example, subsocial aphid *Nipponaphis monzeni* soldiers respond to attacks on their colonies by swarming and exploding their abdomens. Their abdomens are swollen with hemocytes and tyrosine that seal and protect the colony. The endosymbiotic bacterium, *Buchnera*, regulated by aphid host genes, helps overproduce tyrosine (Kutsukake et al., 2019). This example highlights the complex interplay occurring between host, beneficial symbionts, immune system, and social structure of an organism. Other examples of social immunity include grooming, removing waste material and weeding nests and fungal gardens. Further experimentation using antibiotics or probiotics could explore the manner in which microbes may influence behavior and fitness (Alberoni et al., 2018).

Defensive behaviors can also be facilitated by the microbial production of chemical signals or chemical defenses. Social insects participate in extensive grooming behaviors categorized as autogrooming (i.e., self-grooming) and allogrooming (i.e., grooming among nestmates), which serve not only to remove foreign substances from the body surface, but can also provide lasting antimicrobial defenses (Zhukovskaya et al., 2013). In terms of using microbes for production of chemical defenses, many examples in the above defensive symbioses fit this description (e.g., antimicrobial phenols from locust symbionts, antibiotics from fungus-farming ant symbionts). Microbes are also capable of producing chemical signals, such as the intestinal microbes of subterranean termites (*Reticulitermes speratus*), which allow recognition of nestmates from non-nestmate intruders (Matsuura, 2001). The diversity of interactions between defensive microbes and host behavior remains an open area of exploration.

## Human Defenses Against Pathogens

As in insects, the microbiota provides defense against various pathogens in humans, but is more complex than insect microbiomes. While different sites, such as the vagina and nasal cavity can support symbionts with abilities to produce defensive compounds (Donia et al., 2014; Zipperer et al., 2016), most of the potential defensive microbes described reside in the gut. Unlike many insect gut microbiotas, the human gut microbiota may contain hundreds of species (Qin et al., 2010). Adding further complication, whereas in bees and other hosts a core community is evident, a consistent core community has not been identified in humans, although a core functionality appears more conserved than particular strains (Turnbaugh and Gordon, 2009; Human Microbiome Project Consortium, 2012). Although humans lack an equivalent solitary lifestyle to insects, evidence suggests that humans in close social relationships may share a variety of bacteria with one another and have greater richness and diversity than humans living alone (Dill-McFarland et al., 2019).

Many different mechanisms for microbial defense exist and understanding the microbiota’s functions may lead to improved therapies. For example, fecal microbiota transplants for treating *Clostridium difficile* infections that are non-responsive to antibiotics have cure rates of 90% (Bakken et al., 2011; Youngster et al., 2016). Several mechanisms have been suggested including that the microbiota outcompete the pathogen for nutrients, microbially produced antibiotics target *C. difficile*, microbially produced secondary bile acids inhibit *C. difficile*, and microbial interactions with the immune system help repair the gut barrier (Khoruts and Sadowsky, 2016). Human gut microbes have also been linked to defense against *Vibrio cholerae*, where correlations have been found between microbiota taxa present in the gut and resistance to cholera (Hsiao et al., 2014; Midani et al., 2018). Likewise, human microbiota strains compete with *Salmonella* for nutrients and produce metabolites that potentially inhibit *Salmonella* (Antunes et al., 2014; Bratburd et al., 2018; Zhang et al., 2018). Although many interactions and correlations have been suggested between defensive symbiotic bacteria and pathogens in humans, the challenge remains to explore these symbionts on a society-wide scale to understand the benefits not only to individuals but to public health.

Although humans do not have ancient history (on an evolutionary time scale) with agriculture, many crops used by humans associate with defensive microbes against certain pathogens. One example of an agricultural defensive symbiont is *Pseudomonas fluorescens*, a bacterium that produces the antibiotic 2,4-diacetylphloroglucinol, which can inhibit the causative agent of take-all disease in wheat (Keel et al., 1992). This bacterium can be found naturally in soils and is a prominent example of suppressive soils, where soil harbors a community or certain strains that inhibit plant pathogens, analogous to the idea of colonization resistance in animals. Beneficial microbes may provide an environmentally sustainable alternative to chemical control of pathogens and vectors, but will require maintaining beneficial microbes in agricultural settings and consideration of microbial interactions in plant breeding beyond the host’s pathogen resistance (see the following review for more detail (Syed Ab Rahman et al., 2018).

## Interactions of Defensive Symbionts With Host Defenses in Humans

The role of the immune system and behaviors is increasingly recognized as not only defending against harmful microbes, but also fostering the establishment and maintenance of bacterial symbionts. We direct the reader to other reviews for further exploration of the numerous interactions between the microbiota and the immune system (Belkaid and Harrison, 2017) and behavior (Vuong et al., 2017; Johnson and Foster, 2018).

Humans have been practicing their own social immunity with hygienic behaviors throughout history. This includes early ritualistic behaviors, quarantine and sanitation, and after the rise of the germ-theory of disease, water treatment, vaccinations, and vector control (Institute of Medicine (US) Committee for the Study of the Future of Public Health, 1988; Curtis, 2007). While humans have taken advantage of antimicrobial compounds from a variety of sources for hundreds of years (Aminov, 2010; Harrison et al., 2015), large scale antibiotic discovery, often microbially derived, took off in the 1900’s and enabled treating a wide variety of pathogens in people as well as in agriculture (Aminov, 2010). Unfortunately, broad-spectrum antibiotics can have lasting impacts on the microbiota affecting the many interactions discussed above (Jernberg et al., 2007). While efforts to eliminate pathogens have substantial impacts, most notably with vaccines eliminating smallpox and reducing other disease to 99% fewer cases (Orenstein and Ahmed, 2017), practices for sharing beneficial microbes could also be valuable for medicine and agriculture. These practices may include fecal microbiota transplants, probiotic and prebiotic supplementation (George Kerry et al., 2018; Sonnenburg and Sonnenburg, 2019), creating built environments that favor beneficial microbes (Kembel et al., 2012); however, besides perhaps fecal microbiota transplants for treating *C. difficile*, these practices currently lack substantial evidence of efficacy.

## What Can We Learn From Insects?

Insects are useful models to address societal-wide impacts of defensive symbionts (Table 1). Given the vast complexity in the human gut, insects can be a simple model to dissect various mechanisms of microbial defenses since insects tend to have simplified microbiomes relative to humans. Comparisons between social and solitary insects (whether in different life stages as described above with locusts, or among related social and solitary members as described with bees) can shed light on what roles, if any, defensive symbionts have played in the evolution of sociality. Insect colonies are well-defined social units for replication, tend to have limited within colony genetic variation, and can be reared in controlled conditions. The insects themselves often have relatively fast life cycles, which is useful for examining fitness and intergeneration effects defensive microbes may have. Social insects also engage in behaviors of interest, like farming. In the most direct sense, natural products from insect symbioses may be useful as leads for new antibiotics themselves (Stow and Beattie, 2008; Ramadhar et al., 2014; Chevrette et al., 2019) and insects have inherent practical value as many species are important pollinators or pests; however, we also want to highlight using insect models to explore the societal impact of gaining or losing beneficial symbionts. We detailed many benefits of insect models above, but these models come with drawbacks. The simplicities of social insect models limit conclusions relevant for humans to basic ecological dynamics. Insect models lack many features that mediate host-microbe interactions in humans, including an adaptive immune system or complex nervous systems. While much microbiome research has focused on the impact to the individual host, social insects can be used to address basic ecological and evolutionary dynamics including (i) how resilient societies transmit beneficial microbes to other individuals; and (ii) the larger impact of beneficial microbes at the population level.

**TABLE 1:** Comparison of social insect and human models for defensive symbiosis.

Advantages of insect models	Human alternatives
Control of variables (diet, environment, etc.)	Diets and environment generally not experimentally manipulated; metadata may be limited or subject to self-reporting inaccuracies
Defined units of replication for social group (e.g., one colony)	Units could be family, geographical region, etc.
Relatively simple microbiomes	Complex gut microbiomes, other sites varying complexity
Shorter life cycles	Long life cycles
Genetic variation within a colony lower than from a general population	Variable genetic variation
Lifestyle variation exists, including solitary, social, and eusocial members	Different types of social groupings, but all social

Social insect models can address how social animals maximize beneficial microbe transmission while minimizing pathogen spread. Disrupting transmission of beneficial microbes can render hosts more susceptible to disease (Bohnhoff et al., 1954; Currie et al., 1999a; Raymann et al., 2017). In some human societies, transmission and maintenance of microbes has changed dramatically with the introduction of antibiotics, hygiene practices, and diet changes (Bokulich et al., 2016; Vangay et al., 2018). Disruptions in microbiota transmission are hypothesized to have health impacts, including obesity (Principi and Esposito, 2016). In both social insects and humans we have limited understanding of how beneficial microbes are effectively transmitted. In the leaf-cutter ant system, we know that the defensive symbiont *Pseudonocardia* is generally vertically transmitted, acquired during a narrow time window (Marsh et al., 2014) and may use certain host structures (Li et al., 2018), but we do not know what limits bacterial acquisition to certain strains and microbial adaptations to the host. Analogously in humans, we know microbial acquisition begins at birth but the roles and extent of vertically versus horizontally acquired microbes is still debated (Ferretti et al., 2018; Korpela and de Vos, 2018; Moeller et al., 2018; Brito et al., 2019). One drawback of insect models is that specific mechanisms enabling transmission and colonization of beneficial microbes likely differ considerably between insects and humans (e.g., coprophagy is normal behavior for all termite colony members, while fecal microbiota transplant in humans is a medical procedure for the sick). Similarly, humans may travel further and interact with other communities introducing complicated interactions that may not be captured with insect models. However, the defined social structures of eusocial insects may be useful for understanding and manipulating microbial transmission later in life. Reproductive queens have limited contact with other adult workers, for instance, and understanding when and how they share microbes with other castes could illuminate the social elements of microbial transmission (Otani et al., 2019). Microbiomes of distinct nest structures provide an interesting comparison to the idea of built environments (Sharma and Gilbert, 2018).

Additionally, social insect models may address how environmental perturbations such as diet or temperature change the overall community response to pathogens and illuminate fitness effects in different contexts. For example, different substrates used in leafcutter ant fungal gardens impacts overall colony survivorship (Khadempour et al., 2016). While some leafcutter ants associate with defensive symbionts as described above, others rely on their own chemical defenses (Fernández-Marín et al., 2009). The leafcutting ant model could be used to explore how resilient different defensive strategies (chemical or biological control) are to perturbations such as the availability of different substrates. Fisher et al. (2019) predict how other social insect characteristics (including degree of specialization and nest architecture) may enhance susceptibility or resilience to various climate perturbations. The relative simplicity of insect models could help test and reveal basic principles to understand how microbial defenses change in different contexts.

## Conclusion

How societies effectively address risk of pathogen exposure is of increasing concern, especially as the human population size and density rises. Social insects provide a window to explore disease management on a society-wide scale. Increasingly, defensive symbionts are recognized for their valuable role in mitigating pathogens, in insects as well as in humans. Social insects can act as useful models to address the role of defensive symbionts in societies and their interactions with physiological, chemical, and behavioral defenses. Examples from insects provide insight for microbiome-based therapies and agricultural products, as well as help address basic questions on how beneficial microbes are transmitted, maintained, and perturbed in social animals.

## Author Contributions

JB, HH, and RA wrote the manuscript. All authors contributed to the manuscript revision and approved the submitted version.

## Conflict of Interest

The authors declare that the research was conducted in the absence of any commercial or financial relationships that could be construed as a potential conflict of interest.
